# Cancer of the Penis

**Published:** 1900-01-20

**Authors:** 


### CANCER OF THE PENIS.

IN a clinical lecture recently delivered at St. George's Hospital Mr. Marmaduke Sheild[1] drew marked attention to the fact that the great majority of the victims of cancer of the penis have been life-long sufferers from phimosis, and he said that this should be a strong incentive for urging circumcision in the case of children with elongated foreskins. It is, he said, a still more powerful reason in the case of adults who begin to get soreness or irritation beneath a phimosed prepuce, for timely operation may ward off what is one of the most distressing diseases from which a man can suffer. As in all kinds of cancer, early diagnosis is of the utmost importance. The disease in its early beginnings may be either a superficial erosion, a warty growth, or a nodular mass. The "pre-cancerous" or early stage of the disease is marked by so-called "chronic eczema," or by the formation of a warty growth or nodule, and when these conditions, however slight, are associated with phimosis and advancing life the case at once assumes a most anxious aspect. The right course here is at once to advise circumcision, and to apply local antiseptic and soothing remedies in a very dilute form. Above all things the diagnosis must not be complicated by the application of inefficient caustics. The case must be carefully watched, and, once the diagnosis is made, very free removal must be carried out. In regard to diagnosis it must be stated that gummatous ulceration of the penis may simulate cancer, but in this case the base of the sore is devoid of the gristle-like hardness, the margins are serpiginous, and other signs of syphilis will usually be present, as scars on the tongue and legs. Moreover, large doses of iodides soon effect obvious improvement, and should it happen that cancer has engrafted itself upon an old syphilitic sore the sprouting warty mass will soon make its true nature evident. It is otherwise, however, in the diagnosis between primary chancre and cancer. Here extreme caution is necessary. Cases have occurred in which, after amputation has been performed on the diagnosis of cancer, a roseolous rash and subsequent examination has shown that the malady was, in fact, primary syphilis. Great diagnostic difficulties arise in regard to cases of relapsing chancres, in which sores develop *de novo* on the site of former chancres, perhaps as long as 12 months after the original disease, for while cancer may occur in comparatively young men, a man of any age and of any calling may have a hard chancre beneath a phimosed prepuce. In such cases of doubt mercury and iodides should be administered in full doses, we must wait, and before operating a portion of the edge of the sore should be excised under cocaine and examined microscopically. Another diagnostic difficulty may arise between cancer and gummata of the body of the organ, or that rare condition known as "gouty tumour" of the penis, which is of the same nature as Dupuytren's contraction of the palmar fascia, namely, a localised fibrosis leading to thickening and perhaps deformity of the organ. In regard to these it is to be noted that cancer begins on the surface and extends downwards, and that a hard nodule commencing in the corpora can hardly be carcinoma. Sarcoma of the penis is, of course, a possibility, but it is very rare. As to treatment, the difficulty is to get efficient treatment carried out in the early or dubious cases. Take the case of an elderly man with a whitish thickening over the glans, and the formation of an itching wart or horn, with questionable induration at the base. In such cases amputation of the end of the organ is the safer practice. But few patients will submit to such radical treatment for an affection which they consider slight. The alternative is the actual cautery freely used. Never nitric acid or any such inefficient methods. Early removal is then the thing to aim at, and in regard to this removal it is to be remembered that nothing is more miserable than the condition of the man whose urethra opens flush with the pubes. At every effort at micturition urine runs over the scrotum and thighs, which become eczematous and red, and no apparatus can be constructed to carry away the urine from the person. All this is altered, however, if the urethra is made to project from the perineum, as by Gould's operation;[2] in this case the patient when in seclusion micturates like the opposite sex, and when out of doors he uses a receptacle or an apparatus made by Mr. Hawkesley, a sort of glass contrivance, by the use of which the stream is carried away clear of the clothes.

[1] Lancet, Jan. 13. [2] Lancet, May 20, 1882.

